# Early-Life Social Isolation Influences Mouse Ultrasonic Vocalizations during Male-Male Social Encounters

**DOI:** 10.1371/journal.pone.0169705

**Published:** 2017-01-05

**Authors:** Sarah M. Keesom, Caitlyn J. Finton, Gabrielle L. Sell, Laura M. Hurley

**Affiliations:** Department of Biology, Indiana University, Bloomington, Indiana, United States of America; Claremont Colleges, UNITED STATES

## Abstract

Early-life social isolation has profound effects on adult social competence. This is often expressed as increased aggression or inappropriate displays of courtship-related behaviors. The social incompetence exhibited by isolated animals could be in part due to an altered ability to participate in communicatory exchanges. House mice (*Mus musculus*) present an excellent model for exploring this idea, because social isolation has a well-established influence on their social behavior, and mice engage in communication via multiple sensory modalities. Here, we tested the prediction that social isolation during early life would influence ultrasonic vocalizations (USVs) emitted by adult male mice during same-sex social encounters. Starting at three weeks of age, male mice were housed individually or in social groups of four males for five weeks, after which they were placed in one of three types of paired social encounters. Pair types consisted of: two individually housed males, two socially housed males, or an individually housed and a socially housed male (“mixed” pairs). Vocal behavior (USVs) and non-vocal behaviors were recorded from these 15-minute social interactions. Pairs of mice consisting of at least one individually housed male emitted more and longer USVs, with a greater proportional use of USVs containing frequency jumps and 50-kHz components. Individually housed males in the mixed social pairs exhibited increased levels of mounting behavior towards the socially housed males. Mounting in these pairs was positively correlated with increased number and duration of USVs as well as increased proportional use of spectrally more complex USVs. These findings demonstrate that USVs are part of the suite of social behaviors influenced by early-life social isolation, and suggest that altered vocal communication following isolation reflects reduced social competence.

## Introduction

Early-life social experience has dramatic effects on adult behaviors across taxa, including insects [1,2], fish [3,4], reptiles [5], mammals [6,7] and birds [8]. Social experiences during early life can influence an animal’s “social competence”, defined as an animal’s ability to fine-tune its behaviors during social interaction either across or within social contexts [9,10]. Animals raised in a socially impoverished environment can generally be said to express decreased social competence, demonstrating behavioral inflexibility in the social domain. One way in which social incompetence of socially deprived animals is manifested is as an overexpression of aggression. This includes increased displays of aggression at inappropriate times, such as in response to affiliative behaviors [11], or altered use of agonistic signals, yielding more intense aggression from social partners [10,12]. Early-life social experience also shapes social incompetence in the context of courtship and mating. For example, socially impoverished animals exhibit decreased interest in potential mates [13], a lack of a preference between mates of varying quality [14], and a lack of sex preference when performing courtship behaviors, such as vocalization [15,16]. Taken together, these examples suggest that the altered social competence of socially deprived animals could in part be due to changes in their ability to participate in communicatory exchanges, both as receivers and senders of social signals.

The effects of early-life social experience on communication behavior are illustrated by the well-established example of vocal learning by songbirds. Songbirds learn their song from a tutor and will develop abnormal song when they are raised in complete isolation or deafness [17–19]. Although broadcasting a tutor’s song to an isolated juvenile bird can rescue some or all deficiencies in song, the physical presence of social partners also plays an important role in song learning for some species. For example, juvenile birds that are housed together, but isolated from tutor song, will develop song that is intermediate to birds reared with a tutor and birds that were individually isolated [20,21]. Furthermore, same-age cagemates isolated together from a tutor will actually copy their cagemate in preference to broadcast adult song, demonstrating the significance of social partners for some species [22]. Non-singing females can also play a role in shaping male song development through physical and visual signals that reinforce aspects of the learning male’s song [23,24]. In a species in which song learning has been well characterized (zebra finches, *Taeniopygia guttata*), the ability of birds to integrate into a new group (a component of social competence) depends on their social environment during development [8]. Thus, in songbirds, social experience during early life shapes vocal communication, as well as aspects of social competence.

Although house mice (*Mus musculus*) do not learn call production in the same way as songbirds, they provide a compelling system with which to explore the influence of early-life social experience on vocal communication in mammals. First, housing mice in isolation during early life replicates a number of the features characteristic of socially impoverished animals, including elevated aggression [6,7,25]. Additionally, mice are highly social animals that use a variety of communication modalities during social encounters [26]. Ultrasonic vocalizations (USVs) comprise an important component of social signaling for mice [27,28], and furthermore, USVs are sensitive to several types of social experience during adulthood [29–34]. Specifically, one study demonstrated that group-housing versus individual housing of adult mice influences the emission of USVs during encounters with other males [34]. Thus, mice can be used to further investigate the general hypothesis that the social incompetence exhibited by socially deprived animals parallels changes in communication.

Despite a large body of research on the effects of early-life social isolation on other behaviors [6,7,35–38], little is known regarding how early-life social isolation influences adult mouse USVs and whether known non-vocal behavioral changes due to isolation-rearing are accompanied by changes in vocal communication. Here, we addressed these issues by housing 3-week-old male mice either individually or in social groups for four weeks. Following the housing treatment, we staged three different types of dyadic encounters between males to assess how grouped housing versus social isolation influences the use of social USVs. We predicted that socially isolated males would produce more and longer USVs, as described for isolated adults [34]. Furthermore, we predicted that the changes in non-vocal social behaviors would be paralleled by changes in the use of different USV types. The association of distinct types of USVs with particular non-vocal behaviors, such as mounting, allowed us to make an even more specific prediction [34,39–41]. This was that pairs exhibiting increased mounting behavior would exhibit increased proportional use of USVs that have been associated with mounting. Therefore, this study begins to elucidate the roles of social experience versus social isolation in shaping the way in which mice participate as signalers during vocal exchanges.

## Methods

### Animals and housing manipulation

Beginning at post-natal days 18–24, 36 male CBA/J laboratory mice (*Mus musculus*; the Jackson Laboratory, Bar Harbor, ME) were housed individually or in groups of four males, for five weeks. Mice were housed in standard mouse laboratory cages on a 14:10 h light-dark cycle and were provided with water and standard laboratory mouse chow *ad libitum*. Mice remained in their respective housing treatments until the day of staged social encounters. It was unknown whether mice originated from the same litters; however, mice arriving in the same shipment were balanced among different treatment groups. All procedures were approved by the Bloomington Institutional Animal Care and Use Committee (Indiana University, protocol 09–038).

### Staged social encounters

On the day of behavioral testing, home-cages containing the male mice were placed in the experiment room for at least one hour to allow acclimation to the room prior to behavioral recording. All social interactions took place in a neutral arena (a standard laboratory rat cage) lined with clean shavings (Sani-Chip bedding). Two male mice were simultaneously introduced to the arena and allowed to interact freely for the duration of the 15-minute encounter, after which both mice were promptly removed. All interactions were monitored for the presence of fighting behavior, which would have resulted in the mice being separated immediately. However, none of the mice engaged in fighting behavior. In between trials, the testing arena was cleaned with laboratory detergent, wiped down with 70% ethanol, and prepared with clean shavings. Behavioral recordings were made from three pair types (n = 6 pairs per type): two individually housed mice (IND-IND), an individually housed mouse and a socially housed mouse (IND-SOC), and two socially housed mice from different home-cages (SOC-SOC). Mice were marked with 1-cm non-toxic black Sharpie marks on their dorsal pelage for identification during video analysis of behavior. All mice were only used once in the study.

### Behavioral analysis

**Fig 1:**
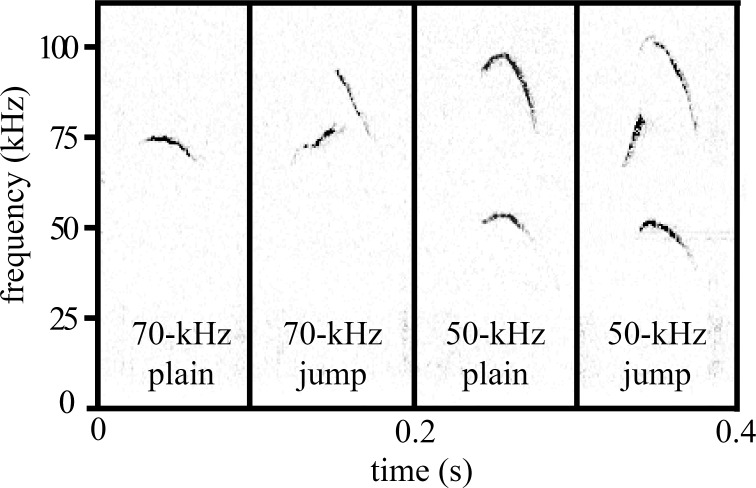
Spectrograms of representative ultrasonic vocalizations (USVs) produced by male mice during same-sex social encounters. USVs were classified based on frequency (50-kHz vs. 70-kHz) and the presence of frequency jumps, yielding four USV types: “70-kHz plain”, “70-kHz jump”, “50-kHz plain”, and “50-kHz jump”. See methods for more details regarding ultrasonic vocalization classification.

For all trials, a charge-coupled device video camera (30 frames/s; Q-See 4-channel DVR PCI video capture card, SuperDVR software, Q-See, Digital Peripheral Solutions) and a condenser microphone (CM16/CMPA; Avisoft Bioacoustics; 200-kHz maximum range) with a sound card (UltraSoundGate 116 Hb; Avisoft Bioacoustics; 250-kHz sample rate) were positioned above the arena. All behavioral analyses were conducted blind to treatment group. Ultrasonic vocalizations were analyzed using spectrograms generated by Avisoft SASlab Pro software (512 FFT-length, Hamming-style window with 50% overlap; Avisoft Bioacoustics). USVs were counted, and the duration of each USV was measured. Past work has characterized USVs based on several features of vocalizations, including frequency[39,42], the presence of an upper harmonic below 120 kHz [40,41], and whether a call includes a jump in frequency [43,44]. Here, we used frequency and the presence of frequency jumps to categorize USVs into four types, defined as follows: “70-kHz plain” (lowest frequency at 70 kHz and no frequency jumps present), “70-kHz jump” (lowest frequency at 70 kHz and jumps in frequency present), “50-kHz plain” (lowest frequency at 50 kHz and harmonic frequency at 100 kHz), and “50-kHz jump” (lowest frequency at 50 kHz, harmonic frequency at 100 kHz, and jumps in frequency present) (Fig 1). We used these categories over a more elaborate classification scheme that has been used by ourselves and others [41,45] because these spectral features are perceptually distinguishable by mice [46]. Although previous studies have assigned individual vocalizations to specific social partners through the use of microphone arrays coupled with motion tracking and discriminatory algorithms [47], the high attenuation rate of ultrasonic sound and the frequent production of USVs when animals are in close proximity to one another (e.g., during social investigation or mounting) makes this a non-trivial task. We thus considered vocalizations as produced by the pair of mice, as have previous studies [34,45,48,49].

Videos of the staged social encounters were analyzed for non-vocal behavior using ODLog (Macropod Software, Eden Prairie, MN). The following social and asocial behaviors were measured for total number and duration: ‘nose-to-nose investigation’, defined as either mouse touching its nose to the other mouse’s facial region; ‘anogenital investigation’, defined as either mouse touching its nose to the area surrounding the base of the other mouse’s tail; ‘mounting’, defined as one of the mice placing its forepaws on the back of the other mouse, typically accompanied by pelvic thrusting; ‘rearing’, defined as a mouse only having its rear legs in contact with the cage floor; and ‘leaping’, defined as a mouse jumping into the air. We also measured ‘locomotion’, defined as the number of times a mouse crossed over a line on a 4 x 2 grid overlaid on top of the video screen. Behaviors were scored together for each pair; however, in the IND-SOC interactions, we identified which mouse of each pair performed the mounting behavior; mounting was consistently performed by the individually housed participant.

### Statistical analysis

All statistical analyses were performed using SPSS 23.0 (IBM). Because our data were not normally distributed, we used nonparametric statistical tests wherever possible. Kruskal-Wallis tests with Bonferroni pairwise comparisons were used to assess differences among social pair types in the total number of USVs, the average duration of USVs, the percent use of different USV types (70-kHz plain, 70-kHz jump, 50-kHz plain, and 50-kHz jump), total duration of nose-to-nose investigation, total duration of anogenital investigation, total number of mounting occurrences, total number of rearing events, total number of leaping events, and total number of lines crossed by a dyad. A two-way ANOVA with Bonferroni pairwise comparisons was used to test for effects of social pair type, USV type, and a pair-type*USV-type interaction on the mean duration of USV types. Because SOC-SOC pairs did not emit all USV types, the two-way ANOVA was conducted on data from IND-IND and IND-SOC pairs only. Spearman rank correlations were used to assess relationships between USVs and non-vocal behaviors. Reported p-values were corrected using the Benjamini-Hochberg [50] method to control for false discovery rate when conducting multiple tests, because this method is an effective strategy for controlling type I errors and reducing the likelihood of type II errors [51].

## Results

### Social housing and pair type influenced vocalizations emitted by male mice

**Fig 2:**
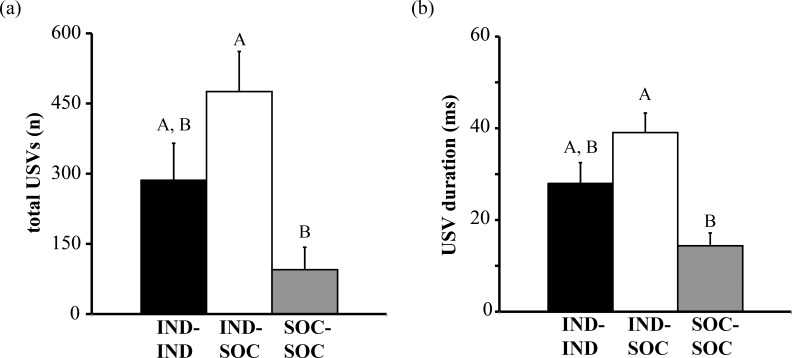
Social history influences the total number and duration of ultrasonic vocalizations (USVs) emitted by male mice. (a) Total number of USVs emitted by pairs of male mice with different social histories. (b) Mean duration of USVs emitted by pairs of male mice with different social housing histories. Bar heights represent means ± S.E.M; bars with different letters are statistically different (Kruskal-Wallis tests with Bonferroni post-hoc tests, p < 0.05). IND = individually housed male; SOC = socially housed male. See methods for additional details regarding housing history.

Here, we tested how male behavior during same-sex encounters is influenced by social experience *via* housing male mice individually or in groups of four. We measured vocal and non-vocal behaviors displayed during 15-min dyadic social interactions in a neutral cage, with three types of social pairs: two individually housed mice (IND-IND), an individually housed mouse and a socially housed mouse (IND-SOC), and two socially housed mice (SOC-SOC). Pair type had a significant effect on many features of USVs. There was a significant effect of pair type on the total number of USVs emitted during an encounter (H = 8.667, df = 2, p = 0.013; Fig 2A), with IND-SOC pairs emitting significantly more USVs than SOC-SOC pairs (p = 0.011) and IND-IND pairs emitting an intermediate number of USVs. The mean duration of USVs also differed by pair type (H = 9.871, df = 2, p = 0.007; Fig 2B): IND-SOC pairs emitted significantly longer duration USVs than SOC-SOC pairs (p = 0.005). USVs emitted by IND-IND pairs had an intermediate mean duration compared with IND-SOC and SOC-SOC pairs.

**Fig 3:**
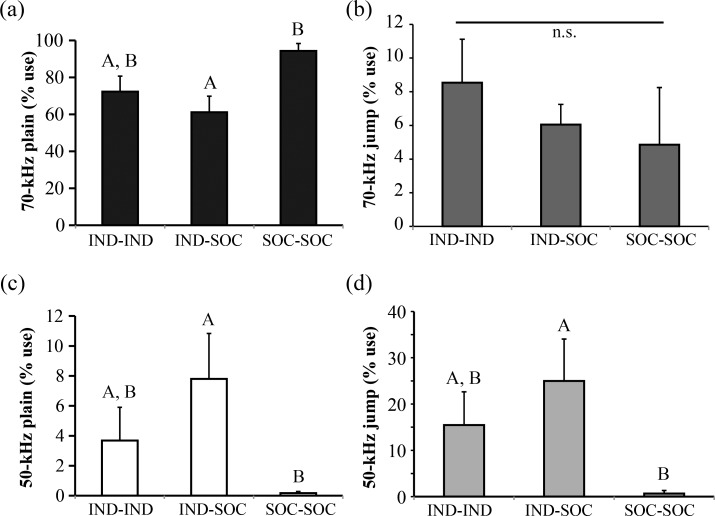
Social history influences the proportional use of different ultrasonic vocalization (USV) types emitted by pairs of male mice. (a) Proportional use of 70-kHz plain USVs. (b) Proportional use of 70-kHz jump USVs. (c) Proportional use of 50-kHz plain USVs. (d) Proportional use of 50-kHz jump USVs. Bar heights represent means ± S.E.M; bars with different letters are statistically different (Kruskal-Wallis tests with Bonferroni post-hoc tests, p < 0.05). IND = individually housed male; SOC = socially housed male. See methods for additional details regarding housing history and USV types.

The proportional usage of USV types also depended on pair type, with a significant effect of pair type on proportional use of 70-kHz plain (H = 7.634, df = 2, p = 0.022; Fig 3A), 50-kHz plain (H = 8.754, df = 2, p = 0.013; Fig 3C), and 50-kHz jump USVs (H = 8.867, df = 2, p = 0.012) (Fig 3D). IND-SOC pairs emitted proportionally more 50-kHz plain and 50-kHz jump USVs than SOC-SOC pairs (p < 0.05), whereas SOC-SOC pairs emitted proportionally more 70-kHz plain USVs than IND-SOC pairs (p = 0.02). IND-IND pairs emitted intermediate proportions of these USV types. All pair types emitted proportionally similar levels of 70-kHz jump USVs (H = 3.700, df = 2, p = 0.157; Fig 3B).

**Fig 4:**
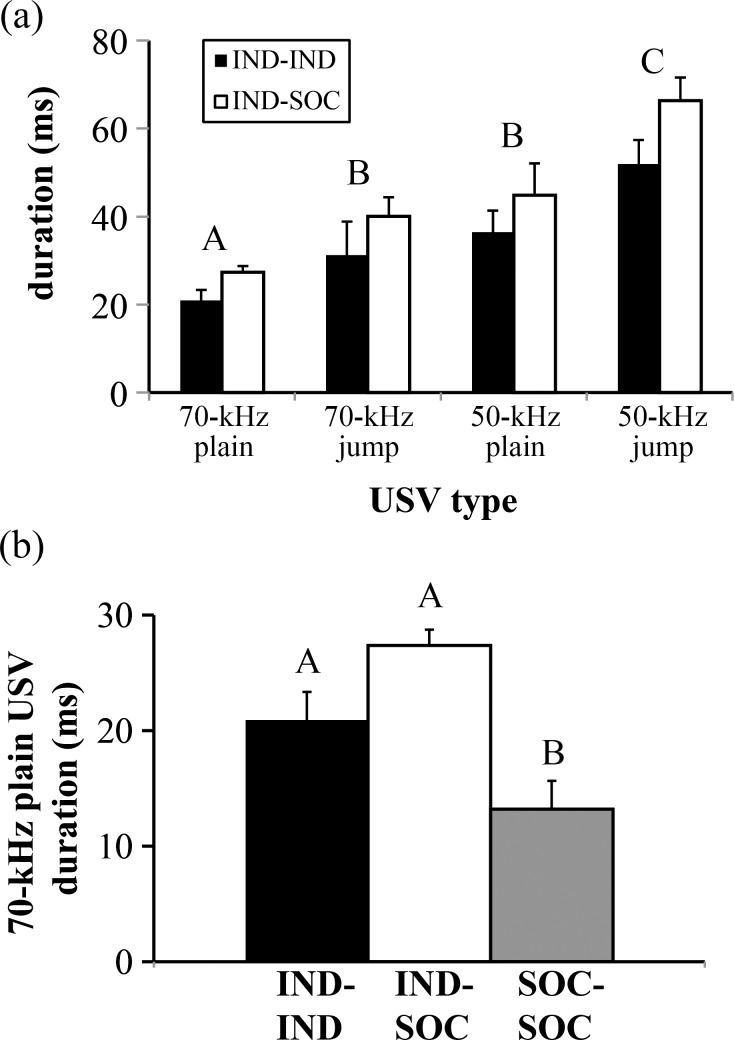
Mean durations of specific USV types emitted by pairs of male mice with different social histories. (a) Mean durations of 70-kHz plain, 70-kHz jump, 50-kHz plain, and 50-kHz jump ultrasonic vocalizations (USVs) emitted by IND-IND and IND-SOC pairs of male mice. SOC-SOC pairs were not included in this two-way analysis, because few SOC-SOC pairs emitted all four USV types. Bar heights represent means ± S.E.M; bars with different letters indicate statistical differences among different USV types (Bonferroni post-hoc tests, p < 0.05). There was also a main effect of pair type: IND-SOC pairs emitted longer duration USVs than IND-IND pairs. (b) Mean duration of 70-kHz plain USVs emitted by pairs of male mice with different social histories. Bar heights represent means ± S.E.M; bars with different letters are statistically different (Bonferroni post-hoc tests, p < 0.05). IND = individually housed male; SOC = socially housed male. See methods for additional details regarding housing history.

The difference among pair types in mean USV duration, as well as in proportional use of particular USV types, raised the possibility that differences in duration among USV types could contribute to differences in mean USV duration among the three pair types. Thus, we investigated durations of USV types emitted by IND-IND and IND-SOC pairs, which emitted all four USV types. SOC-SOC pairs were not included in this two-way analysis, because few SOC-SOC pairs emitted all four USV types. For IND-IND and IND-SOC pairs, the four USV types were significantly different in duration (F_3,38_ = 15.295, p < 0.001; Fig 4A). 50-kHz jump USVs were significantly longer than all three other USV types (p < 0.01), 50-kHz plain USVs were significantly longer than 70-kHz plain USVs (p = 0.02), and 70-kHz jump USVs were intermediate in duration to 50-kHz plain and 70-kHz plain USVs. Additionally, there was a main effect of pair type on USV duration across USV types (F_1,38_ = 6.584, p = 0.014), with no interaction between pair type and USV type (F_3,38_ = 0.207, p = 0.891). Thus, even within USV type, IND-SOC pairs had consistently longer-duration USVs than IND-IND pairs. This pattern is also demonstrated by examining the duration of 70-kHz plain USVs (emitted by all pair types) across the three pair types. There was a significant effect of pair type of the duration of 70-kHz plain USVs (F_2,9.168_ = 12.380, p = 0.002; Fig 4B), with SOC-SOC pairs emitting significantly shorter 70-kHz plain USVs than IND-IND pairs (p = 0.02) and IND-SOC pairs (p = 0.001).

### Social housing and pair type influenced non-vocal social behaviors

**Fig 5:**
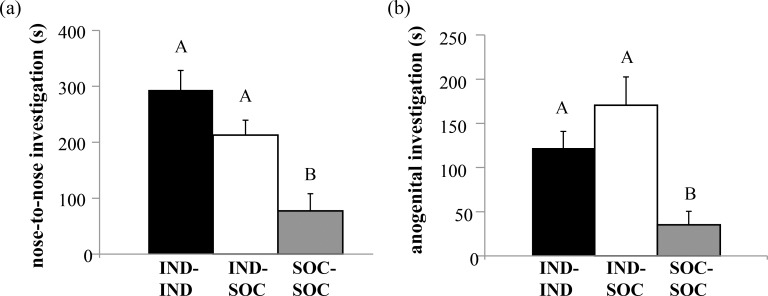
Social history influenced the total durations of social investigation behaviors performed by pairs of male mice. (a) Nose-to-nose investigation exhibited by pairs of male mice with different social histories. (b) Anogenital investigation exhibited by pairs of male mice with different social histories. Bar heights represent means ± S.E.M; bars with different letters are statistically different (Kruskal-Wallis tests with Bonferroni post-hoc tests, p < 0.05). IND = individually housed male; SOC = socially housed male. See methods for additional details regarding housing history.

**Fig 6:**
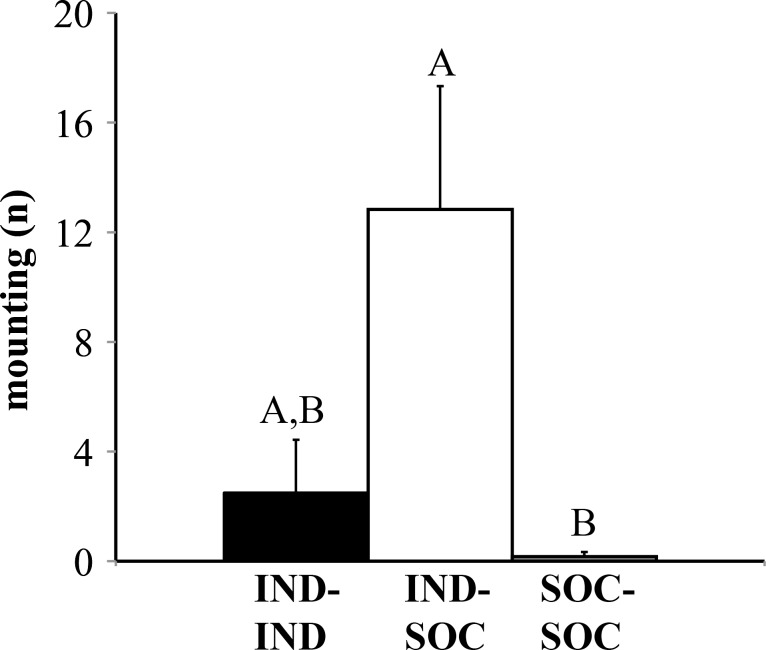
Number of mounting occurrences performed by pairs of male mice with different social histories. For IND-SOC pairs, IND males mounted SOC males. Bar heights represent means ± S.E.M; bars with different letters are statistically different (Kruskal-Wallis test with Bonferroni post-hoc tests, p < 0.05). IND = individually housed male; SOC = socially housed male. See methods for additional details regarding housing history.

Non-vocal behaviors were also affected by pair type. There was a significant effect of social housing on nose-to-nose investigation (H = 10.959, df = 2, p = 0.004; Fig 5A) and anogenital investigation time (H = 9.556, df = 2, p = 0.008; Fig 5B). Corroborating previous literature that reported increased social investigation in individually housed animals [6,25], IND-IND and IND-SOC pairs performed more nose-to-nose and anogenital investigation than SOC-SOC pairs (p < 0.05). IND-IND and IND-SOC pairs displayed statistically similar levels of nose-to-nose and anogenital investigation (p > 0.05). Pair type also had a significant effect on the number of mounts exhibited (H = 9.844, df = 2, p = 0.007; Fig 6), with significantly more mounts displayed by IND-SOC pairs than SOC-SOC pairs (p = 0.006). IND-IND pairs displayed an intermediate amount of mounting. It is important to note that during interactions between IND-SOC pairs, it was the IND mouse that consistently performed mounting behavior towards the SOC mouse. In contrast to these differences by pair type in social behaviors, pair types displayed similar levels of non-social behaviors, including rearing, leaping, and locomotion (Kruskal-Wallis tests, p > 0.05).

### Relationships between vocal and non-vocal behaviors

**Fig 7:**
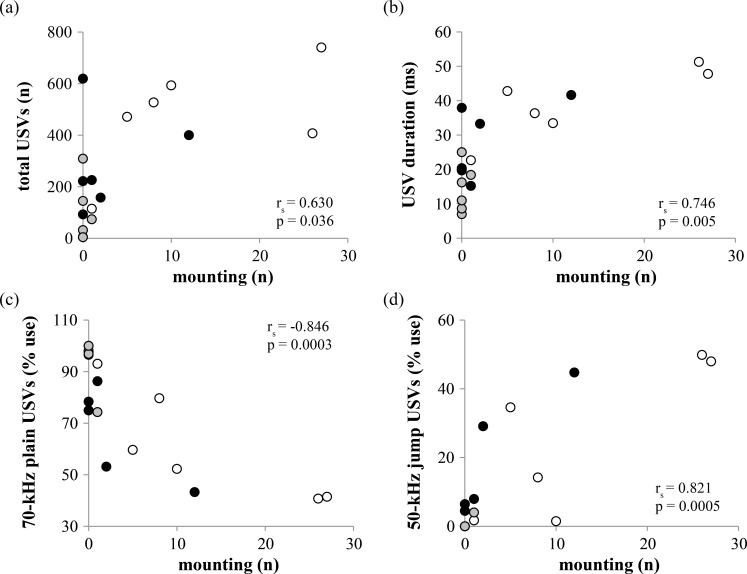
Mounting behavior is correlated with several aspects of ultrasonic vocalizations emitted by pairs of male mice with different social histories. (a) Mounting versus total ultrasonic vocalizations (USVs). (b) Mounting versus USV duration. (c) Mounting versus proportional use of 70-kHz plain USVs. (d) Mounting versus proportional use of 50-kHz jump USVs. Black circles: IND-IND pairs; white circles: IND-SOC pairs; gray circles: SOC-SOC pairs. IND = individually housed male; SOC = socially housed male. See methods for additional details regarding housing history.

We assessed how multiple qualities of ultrasonic vocalizations were related to non-vocal behaviors across different interactions between unfamiliar mice. Mounting behavior was significantly related to several characteristics of USVs: total number (r_s_ = 0.630; p = 0.036; Fig 7A), average duration of USVs (r_s_ = 0.746, p = 0.005; Fig 7B), proportional use of 70-kHz plain USVs (r_s_ = -0.846, p = 0.0003; Fig 7C), and proportional use of 50-kHz jump USVs (r_s_ = 0.821, p = 0.0005; Fig 7D). Thus, interactions between males that displayed more mounting behavior also emitted more USVs that were longer duration, consisting of proportionally more 50-kHz jump USVs and proportionally fewer 70-kHz plain USVs. These patterns were also reflected within pair types that included at least one individually housed male. For instance, mounting was significantly correlated with proportional use of 50-kHz jump USVs across IND-IND pairs (r_s_ = 0.941, p = 0.005). Mounting was significantly, negatively correlated with proportional use of 70-kHz plain USVs across IND-SOC pairs (r_s_ = -0.886, p = 0.019) (and thus, mounting of SOC mice by IND mice was positively associated with USVs of relatively greater spectral complexity for these pairs). Correlations between mounting and vocal behavior were not observed within SOC-SOC familiar pairs, likely due to the low occurrence of mounting for this pair type. None of the other social or non-social behaviors we measured were related to the quantity or any qualities of USVs we measured (p > 0.05 for all).

## Discussion

Social experience has a profound influence on vocal communication across species. Here, we tested the prediction that social isolation during early life would influence vocal signals of adult male mice during same-sex encounters. More and longer USVs were emitted by pairs including at least one individually housed male (IND-IND and IND-SOC pairs), compared to pairs of two socially housed males (SOC-SOC pairs). Furthermore, the social history of pairs influenced the proportional use of different types of USVs. In particular, pairs of group-housed males emitted USVs that were almost entirely composed of 70-kHz plain USVs. In contrast, proportionally more 50-kHz plain and 50-kHz jump USVs were emitted by pairs that included at least one individually housed male. In accordance with the literature on the effects of social isolation [6,7], pairs that included individually housed mice also engaged in an elevated degree of social investigation, including both nose-to-nose investigation and anogenital investigation. Interestingly, individually housed mice, during the IND-SOC pair interactions, exhibited increased mounting behavior toward the socially housed mice relative to IND-IND and SOC-SOC pairs, and mounting behavior was positively correlated with several aspects of USVs. In contrast, non-social behaviors, including rearing, leaping, and locomotion, were unaffected by isolation. Thus, these results demonstrate that vocal signals are part of a suite of mouse social behavior influenced by social isolation in early life. In this discussion, we will consider our findings as they relate to the functional significance of USV subcategories, as they relate to the hypothesis that social experience influences social competence, as they relate to changes in socially induced behavioral arousal, and as they relate to the idea that social behavior is an emergent property of social groups.

### Functional significance of call categories

Here, we classified USVs into four categories, in contrast to more extensive categorization schemes that we and others have used in the past [41,45]. Our “50-kHz plain” and “50-kHz jump” USVs were distinguished from the other two by the presence of a clear upper harmonic element. Although harmonics may have existed beyond our range of measurement for the 70-kHz calls, there are additional reasons to categorize the 70-kHz and 50-kHz USVs separately. One of these is that frequency is an important and sometimes the exclusive criterion for call categorization in rodents. In mice, ultrasonic calls made by males have been separated strictly by frequency, into 70-kHz and 40-kHz USVs [39,52], likely corresponding to our “70-kHz” versus “50-kHz” USVs. In laboratory rats (*Rattus norvegicus*), “22-kHz” versus “50-kHz” calls are a common distinction, although 50-kHz calls have more recently been sub-classified based on spectral structure [53,54].

These frequency categories of vocalizations in rats and mice are also associated with distinct behaviors. In rats, “22-kHz” calls are associated with aversive behaviors, while “50-kHz” calls are generally regarded as prosocial [55,56]. For mice, “70-kHz” and “40-kHz” USVs have both been associated with courtship by males, but “40-kHz” USVs are more exclusively associated with mounting [39]. Likewise, we and others have found that USVs incorporating a lower-frequency harmonic (“harmonic” calls in [41]; here, “50-kHz” calls) closely correspond to mounting behavior [41,57]. In contrast, calls with the lowest frequency of near 70 kHz are not closely associated with mounting [41]. There is also a recently discovered ‘mid-frequency’ call of mice with a fundamental frequency of around 12 kHz that has been associated with distress [58]. The difference between our 50-kHz and 70-kHz categories therefore does not simply represent a limitation on our measurement, but a functional distinction.

In general, these functional distinctions have been established for the calls of male mice interacting with females. USVs produced by male house mice have often been viewed in the context of courtship, since they are androgen-sensitive [59] and are triggered more strongly by female cues than by those of males in some studies [60,61]. Despite the predominance of male-female studies of mouse vocalization patterns, multiple studies have documented male mice producing USVs during male-male encounters, using a variety of USV types [34,45,60–62]. Generally, USVs emitted during male-male social encounters are associated with social investigation and occur prior to any fighting behavior [34,45,60]; however, there is little information regarding how specific types of USVs relate to specific social behaviors in male-male interactions. The association between mounting and specific call types in the current study represents an advance in this area, and a similarity in male USVs used during same-sex encounters and male USVs used during male-female encounters.

### Influences of social experience on social competence

Social competence is defined as the ability of an animal to modify its social behaviors according to current social information [9,10]. In other words, a socially competent animal will engage in “appropriate” behavior during social interaction in two ways: 1) A socially competent animal will display behavior that is appropriate to the general context, for example, by using courtship behaviors exclusively during mixed-sex contexts. 2) An animal can also demonstrate social competence by displaying behavior that is graded to the particular social situation, for example, by initiating aggression with threat displays instead of full attacks if a social partner is not exhibiting aggressive behavior.

By these criteria, one possible interpretation of our findings is that the individually housed male mice inappropriately displayed courtship and mating behavior toward same-sex conspecifics. Support for this interpretation is based on two assumptions. The first of these is that high levels of vocalization, including usage of USVs with frequency jumps and lower frequency (40–50 kHz) elements, are only appropriately emitted by males toward females. Evidence for this view is that males emit copious vocalizations when placed with females, and that vocalizations with lower-frequency components are associated in time and number with male mounting [41,57]. Furthermore, USVs including lower frequency (40–50 kHz) elements and frequency jumps are more attractive to female mice compared to USVs without these features [63]. The increasing numbers of studies reporting vocalizations between male-male and female-female mouse pairs suggests that vocalizations serve a social signaling role outside of opposite-sex interaction, however [45,61,62,64].

A second assumption is that mounting is a behavior only appropriately displayed by males towards females [65,66]. This assumption is supported by studies that disrupted the functionality of the vomeronasal organ (an organ that detects pheromonal cues, such as those from females). These studies found that male mice with lesioned vomeronasal organs vocalized to and mounted other males in equal amounts as towards female mice, whereas males with intact vomeronasal organs directed these behaviors exclusively towards females [65,66]. The authors interpreted the expression of copious USVs and mounting as purely sexual behavior, and thus, supportive of males with disrupted vomeronasal organs being unable to discriminate between the sexes, instead of these males displaying dominance behavior [66]. In another study, male mice without vasopression 1b receptors displayed little attack behavior, but elevated mounting towards other males, as opposed to wild-type mice that displayed stereotypical aggressive behaviors (consisting of attacks towards other males and little mounting) [67]. This finding was interpreted as Avpr1b knock-out mice using a “different strategy” for establishing dominance, although the authors concede that the lack of Avpr1b could also have affected the animal’s sense of olfaction and thus, the knock-out mice may have been exhibiting a more general problem of displaying inappropriate social behavior, similar to the findings and interpretation by Stowers et al. [66].

However, mounting behavior has also been interpreted as reflecting dominance among male mice. This is demonstrated by smaller, subordinate males displaying less mounting, fighting, and attacking, than larger, dominant mice [68]. In our study, if mounting was indicative of aggressive rather than misdirected sexual behavior, then mice that were housed individually exhibited greater levels of aggression than socially housed mice. Instead of representing the use of a contextually inappropriate social behavior, increased mounting and vocalizing by previously isolated mice could therefore represent a behavioral performance that is not graded to the social situation. In particular, high levels of mounting were observed in IND-SOC pairs, and it was the individually housed male that displayed mounting toward the socially housed male. Both IND-IND and IND-SOC pairs showed high levels of vocalization, with the use of 50-kHz jump USVs positively related to mounting behavior. Instead of being indicative of sexual behavior, this type of vocalization could simply be related to the non-vocal behaviors expressed by individually housed mice. Because IND mice in the IND-SOC pairs showed high levels of mounting and vocalization whereas SOC-SOC pairs showed relatively low levels of these behaviors, one interpretation of these data is that previously isolated mice were not able to distinguish between relatively nonaggressive and aggressive partners. This conclusion would fit the second definition of social competence, in which socially incompetent individuals do not grade their behavior in a way appropriate to a given situation.

The idea that social experience allows animals to appropriately grade aggressive behavior is supported across taxa. For example, animals that have gained social experience via group-living employ strategies other than actual fighting, such as aggressive or submissive signals, when encountering new individuals [69,70]. In contrast, animals that are raised in an impoverished social environment, as opposed to being raised in larger or more complex groups, typically rely solely on physical aggression, and are therefore less able to interact peacefully with new conspecifics, a phenomenon that is demonstrated in fish [69], birds [8], rodents [12,70,71], and primates [11]. For example, socially deprived female rhesus macques demonstrate increased aggressive behavior in response to grooming attempts by novel social partners [11]. This example illustrates a display of aggressive behavior that is *not* appropriately graded, given that grooming is an affiliative behavior in macaques, and therefore does not pose a threat to an animal’s survival.

### Ultrasonic vocalizations as a read-out of increased behavioral arousal in individually housed mice

Contributing to effects of experience on social vocal behavior could be effects of experience on socially induced behavioral arousal. For example, the increased social investigation exhibited by IND mice compared to SOC mice is consistent with increased socially induced behavioral arousal in IND mice. In this alternative interpretation, the longer duration USVs, comprised of more 50-kHz calls, emitted by IND males could simply be a reflection of increased social arousal in IND mice compared to SOC mice. This interpretation is consistent with studies in which USVs were compared between highly social strains of mice and a strain of mice that was specifically bred to be less socially active (BTBR strain, an autistic mouse model). In BTBR mice, their decreased level of social investigation coincides with decreased emission of USVs compared to strains that are highly social [45,72], a result that is comparable to the current study, in which SOC mice emitted few USVs and displayed decreased social investigation. Furthermore, the less socially aroused BTBR mice emit proportionally fewer USV types that contain frequency jumps, similar to our finding that pairs of SOC males emit significantly fewer of these spectrally more complex calls [45].

Experimental manipulations of stimuli that induce varying degrees of behavioral arousal also alter USV emission, in ways that are similar to the USVs emitted by the more socially aroused individually housed mice in the current study. For example, one experiment presented male mice with a novel stimulus (cotton pad), female olfactory cues, and an actual female partner, stimuli that induce consecutively increased behavioral arousal in male mice [73]. Ultrasonic vocalization paralleled increased behavioral arousal, with males emitting more USVs in response to female olfactory cues than the cotton pad, and more USVs in response to the presence of a female partner compared to just female odors [73]. In another experiment, the intensity of the female olfactory cue was manipulated, and the number of USVs emitted by male mice in response to female odor was positively related to the concentration of female urine [74]. Finally, one study manipulated the behavioral state of the social partner (anesthetized partner versus vivid partner) [61]. Given that a vivid partner would be reciprocally interacting with the subject animal, this would likely induce a state of higher behavioral arousal than an anesthetized partner. In response to the potentially more behaviorally arousing vivid partner, male mice emitted more and longer duration USVs, with greater proportional use of USVs containing frequency jumps [61]. Thus, mice that experience more behaviorally arousing situations, such as more arousing social stimuli, emit more and longer duration USVs with increased spectral complexity. This pattern of calling is similar to the pattern demonstrated by IND mice in the current study, suggesting that the USVs produced IND mice are in part a result of increased behavioral arousal during social situations.

### Social behavior as an emergent property

In this study, the largest difference in social behavior was generally found between IND-SOC pairs and SOC-SOC pairs. This is interesting because one might predict that IND-SOC pairs would display intermediate levels of social behavior, and that the greatest difference in behavior would be between the IND-IND and SOC-SOC pairs. However, social behavior exhibited by a dyad or group of animals is not necessarily the summed behavior of the participants. Behaviors of one social participant depend on the behaviors exhibited by other participants [75–77], and thus, social behavior at the group level can be regarded as an emergent property [78–80]. In our study, this phenomenon is best illustrated by how pairs exhibited mounting behavior. IND-SOC pairs displayed the highest level of mounting, which was consistently performed by the IND mouse towards the SOC mouse. If the social behaviors exhibited by a pair of mice were additive, one would expect IND-IND pairs to exhibit more mounting behavior compared to IND-SOC pairs, and SOC-SOC pairs to display the lowest level of mounting behavior. While the prediction for SOC-SOC pairs holds true, mounting behavior displayed by IND-IND pairs was intermediate to IND-SOC and SOC-SOC pairs. A potential explanation of this result lies in the behavioral phenotypes of individually housed animals compared to socially housed animals. Individually housed animals display more aggression and dominance behaviors [6,10,12], along with increased reactivity to social contact [11], whereas socially housed animals exhibit positive responses to social contact [11,70]. Therefore, one possible interpretation of the highest mounting being exhibited by IND-SOC pairs is that the SOC mice permit mounting behavior performed by IND mice, whereas mounting attempts made by IND mice are inhibited during the IND-IND pairings, due to the high reactivity of the other IND social partner. Our findings therefore provide a putative example of how differing past experiences of individual animals contribute to the emergence of suites of social behavior used during dyadic social interactions.

## Supporting Information

S1 Excel Data File containing the USV data and non-vocal behavioral data reported in this paper. (XLSX)
